# Distemper, extinction, and vaccination of the Amur tiger

**DOI:** 10.1073/pnas.2000153117

**Published:** 2020-11-23

**Authors:** Martin Gilbert, Nadezhda Sulikhan, Olga Uphyrkina, Mikhail Goncharuk, Linda Kerley, Enrique Hernandez Castro, Richard Reeve, Tracie Seimon, Denise McAloose, Ivan V. Seryodkin, Sergey V. Naidenko, Christopher A. Davis, Gavin S. Wilkie, Sreenu B. Vattipally, Walt E. Adamson, Chris Hinds, Emma C. Thomson, Brian J. Willett, Margaret J. Hosie, Nicola Logan, Michael McDonald, Robert J. Ossiboff, Elena I. Shevtsova, Stepan Belyakin, Anna A. Yurlova, Steven A. Osofsky, Dale G. Miquelle, Louise Matthews, Sarah Cleaveland

**Affiliations:** ^a^Cornell Wildlife Health Center, College of Veterinary Medicine, Cornell University, Ithaca, NY 14853;; ^b^Boyd Orr Centre for Population and Ecosystem Health, Institute of Biodiversity Animal Health and Comparative Medicine, University of Glasgow, Glasgow G12 8QQ, United Kingdom;; ^c^Wildlife Conservation Society, Bronx, NY 10460;; ^d^Federal Scientific Center of the East Asia Terrestrial Biodiversity, Far Eastern Branch of Russian Academy of Sciences, Vladivostok 690022, Russia;; ^e^Land of the Leopard National Park, Vladivostok 690068, Russia;; ^f^Zoological Society of London, London NW1 4RY, United Kingdom;; ^g^Primorskaya State Agricultural Academy, Ussuriisk 692510, Russia;; ^h^United Administration of Lazovsky Zapovednik and Zov Tigra National Park, Lazo 692890, Russia;; ^i^Autonomous Noncommercial Organization “Amur,” Lazo 692890, Russia;; ^j^Pacific Geographical Institute, Far Eastern Branch of the Russian Academy of Sciences, Vladivostok 690041, Russia;; ^k^Far Eastern Federal University, Vladivostok 690091 Russia;; ^l^A. N. Severtsov Institute of Ecology and Evolution, Russian Academy of Sciences, Moscow 119071, Russia;; ^m^Medical Research Council–University of Glasgow Centre for Virus Research, Glasgow G61 1QH, United Kingdom;; ^n^Department of Comparative, Diagnostic, and Population Medicine, College of Veterinary Medicine, University of Florida, Gainesville, FL 32610;; ^o^Institute of Molecular and Cellular Biology, Siberian Branch of the Russian Academy of Sciences, Novosibirsk 630090, Russia

**Keywords:** Amur tiger, *Panthera tigris altaica*, canine distemper virus, wildlife vaccination, extinction

## Abstract

Canine distemper virus (CDV) has recently emerged as an extinction threat for the endangered Amur tiger (*Panthera tigris altaica*). CDV is vaccine-preventable, and control strategies could require vaccination of domestic dogs and/or wildlife populations. However, vaccination of endangered wildlife remains controversial, which has led to a focus on interventions in domestic dogs, often assumed to be the source of infection. Effective decision making requires an understanding of the true reservoir dynamics, which poses substantial challenges in remote areas with diverse host communities. We carried out serological, demographic, and phylogenetic studies of dog and wildlife populations in the Russian Far East to show that a number of wildlife species are more important than dogs, both in maintaining CDV and as sources of infection for tigers. Critically, therefore, because CDV circulates among multiple wildlife sources, dog vaccination alone would not be effective at protecting tigers. We show, however, that low-coverage vaccination of tigers themselves is feasible and would produce substantive reductions in extinction risks. Vaccination of endangered wildlife provides a valuable component of conservation strategies for endangered species.

## Significance

The decline and progressive fragmentation of many threatened populations increase extinction vulnerability due to outbreaks of infectious disease. Vaccination is one of the few tools available to mitigate these threats, but its use is often hampered by insufficient epidemiological understanding and historic controversies over endangered wildlife vaccination. Using the example of Amur tigers and CDV, we describe a holistic approach to select appropriate disease mitigation strategies based on key epidemiological evidence from the field. We then assess the protection of vaccinated tigers against the locally circulating CDV strain and use modeling to compare the efficacy and cost of potential vaccination programs. This practical approach provides conservation managers with an evidence-based rationale to address disease-mediated extinction risks for threatened wildlife populations.

Tigers (*Panthera tigris*), among the world’s most iconic carnivore species, are highly threatened. Having once occupied vast swathes of Asia from Turkey to the Sea of Japan, fewer than 3,500 tigers now survive with breeding populations in just eight countries, all of which are fragmented and acutely vulnerable to extinction (1). The Amur tiger subspecies (*P. tigris altaica*) numbers fewer than 550 individuals in two discrete populations in the Russian Far East and neighboring areas of China.

## Results and Discussion

Infectious diseases are increasingly recognized as an extinction threat for endangered carnivores, and viral pathogens, particularly those linked with domestic dogs (*Canis familiaris*), have been the cause of major declines in several populations (2). One of these pathogens, canine distemper virus (CDV) (*Canine morbillivirus*), is commonly associated with domestic dogs but has also caused disease outbreaks in Serengeti lions (*Panthera leo*), Ethiopian wolves (*Canis simensis*), and Channel Island foxes (*Urocyon littoralis*) (3–5), and is now also emerging as a threat to Amur tigers. CDV was first detected as the cause of death in Amur tigers in 2003, with subsequent cases confirmed in 2010 (6, 7); population viability analyses showed that CDV increased the 50-y extinction probability of small populations to over 50%, an increase of up to 65% (8). These findings have highlighted the need for active management of disease in the conservation of the Amur tiger and possibly other more fragmented tiger populations across the species’ range.

Several approaches to disease management might be considered, but effective decision making requires both an understanding of which host species act as sources and reservoirs of infection (9) and a feasibility assessment of potential interventions. For CDV in the Russian Far East, investigation of reservoirs and implementation of interventions both pose a substantial challenge. CDV can infect many host species, with the Amur tiger habitat supporting highly diverse carnivore communities (17 wild carnivore species along with domestic dogs) and these areas span large, remote tracts of land.

Disease interventions could be designed to reduce transmission to tigers through blocking tactics (e.g., limiting contact between tigers and potential source populations such as domestic dogs or other wild carnivores), or by reducing CDV infection prevalence in reservoir or source populations (e.g., through vaccination). Alternatively, vaccination could be directed to the target population of primary concern, in this case tigers. However, vaccination of endangered wildlife populations has in the past been beset by controversy (10–12), and this has led conservation managers to focus primarily on vaccination of domestic dogs—established as important sources of infection in other ecosystems (13, 14). Indeed, conservationists and the general public often assume domestic dogs must be the source of CDV infection for endangered wildlife (15, 16).

Our first objective was to generate and evaluate epidemiological evidence to determine the likely relative importance of domestic dogs and wild carnivore hosts as sources of CDV infection for Amur tigers in the Primorskii Krai region of Russia. The challenges of sample collection typical of work in such remote regions required us to draw on multiple sources of evidence to unravel reservoir relationships. These include CDV serological data, which provides a reliable indicator of prior infection but has limited power to discern precise temporal patterns, and virus sequence data, which has the potential to generate powerful insights but is hampered by the short duration of CDV infectivity that limits opportunities for virus detection or isolation.

The first confirmed case of CDV infection was in 2003, and a retrospective analysis of serological data in Amur tigers supports the contention that CDV was recently introduced into this population. CDV antibodies were not detected in any of 18 tigers sampled prior to 2000 but were detected in 20 of 54 tigers (37.0%) sampled since then (Table 1).

**Table 1.** Summary of virus neutralization results against CDV from large carnivores sampled in the Russian Far East from 1992 to 1999, and from 2000 to 2014

	Animals sampled during 1992 to 1999				Animals sampled during 2000 to 2014			
Species	+ve	*N*	%	95% CI	+ve	*n*	%	95% CI
Amur tiger	0	18	0.0	0–21.9	20	54	37.0	24.6–51.37
Far Eastern leopard	2	6	33.3	6.0–75.9	0	4	0.0	0.0–60.4
Eurasian lynx	0	0	—	—	1	7	14.3	0.8–58.0
Asiatic black bear	0	9	0.0	0.0–37.1	1	17	5.9	0.3–30.8
Brown bear	1	13	7.7	0.4–37.9	1	8	12.5	0.7–53.3
Total	3	46	6.5	1.7–18.9	23	90	25.6	17.2–36.0

For animals sampled on more than one occasion, only the most recent sample in each period is included. Samples were analyzed by Washington State University using the Onderstepoort strain of CDV.

Serological and demographic data also provide insight on the role of domestic dogs and other wildlife. CDV antibodies were detected in unvaccinated dogs from 24 of 37 settlements sampled and in eight wild carnivore species from eight locations across Primorskii Krai, confirming that CDV exposure was widespread (*SI Appendix*, Fig. S2 and Table S1). Contrary to expectations, we detected a higher CDV seroprevalence and higher frequency of CDV outbreaks among dogs in small remote populations than in more densely populated areas (Table 2 and *SI Appendix*, Fig. S2). Of the 32 dogs in the most remote survey community, all had been born there, and none had traveled to other settlements; nevertheless we detected antibodies in four of the five unvaccinated dogs sampled, including a 12-mo-old pup. This population is far too small to maintain the pathogen (17), so the most plausible explanation for recently infected individuals is that wildlife constitutes an important source of CDV infection for dogs, challenging a widely held view that the epidemiological cycle is dominated by dog-to-wildlife transmission.

**Table 2.** Results of virus neutralization analyses against CDV for serum samples collected from unvaccinated dogs in the study sites LLNP, Lazovskii Zapovednik, and SABZ

Study area	Total no. of settlements	Dog density, dogs⋅km^2^	No. of settlements sampled	No. of settlements with recent outbreaks (*n*)	No. of positive dogs (*n*)	Seroprevalence (95% CI)
LLNP	64	2.5	27	4 (19)	29 (182)	15.9 (11.1–22.3)
Lazovskii	15	1.1	7	3 (6)	49 (166)	29.5 (22.8–37.2)
SABZ	4	0.3	3	3 (3)*	48 (116)	41.4 (32.4–50.9)

Dog densities were based on extrapolation of human/dog ratios from questionnaire surveys. Neutralizing antibody titers of 1:16 or higher were considered positive. Seroprevalence is given as the number of positive samples expressed as a percentage of sample size, with lower and upper 95% binomial CIs. Positives in dogs aged 4 to 12 mo were used to identify recent outbreaks (note that this age class was not represented in all communities). Samples were tested at the University of Glasgow using the Onderstepoort strain of CDV [cell culture adapted by Bussell and Karzon (18)].

* Denotes that recent outbreaks were detected in one of these communities during surveys in 2012 and 2014.

Patterns of dog movement and population connectivity raise further questions about the role of domestic dogs as a potential maintenance population. The estimated size of the dog population across Primorskii Krai as a whole (467,244 CI: 442,549 to 496,933 dogs) may be large enough to exceed the critical community size needed to maintain a morbillivirus infection (compared to an estimated combined population of the of the four most abundant wild carnivore species of between 196,850 and 585,900 animals; *SI Appendix*, Table S2). However, the relationship between host population size and CDV persistence is likely to be modified by the limited connectivity between subpopulations. Survey data indicated that only 6.1% of dogs were permitted complete freedom of movement, and only 6.9% were ever taken to other settlements, providing few opportunities for CDV transmission (17).

Analysis of genetic sequence data provides further support for wildlife acting as a reservoir (*SI Appendix*, Tables S3 and S4). For tigers, sequence data were obtained from a combination of live and postmortem sampling events conducted between 2000 and 2014 [*n* = 23 tigers, including three known CDV cases from 2003 and 2010 (7)]. We obtained sequence data corresponding to the hemagglutinin attachment glycoprotein (H-gene, 1,824 bp) from two previously unidentified cases involving tigers which died in 2006 (GenBank accession no. KX708720) and in 2013 (KX708726). We also obtained 98.83% of the CDV genome (15,690 bp) from a formalin-fixed paraffin-embedded brain specimen from the 2003 case (KX774415). From other wildlife, full H-genes were sequenced from 22 wild carnivores, with viruses detected in each of the 4 y sampled (2012 to 2015; *SI Appendix*, Tables S3 and S4), and a further sequence from a critically endangered Far Eastern leopard (*Panthera pardus orientalis*) in 2015 (MK169401). Despite extensive sampling, only one partial H-gene sequence was obtained from a domestic dog. This sample was collected during a CDV outbreak in Vladivostok in 2016 (539 bp, MK169402).

**Fig. 1.**
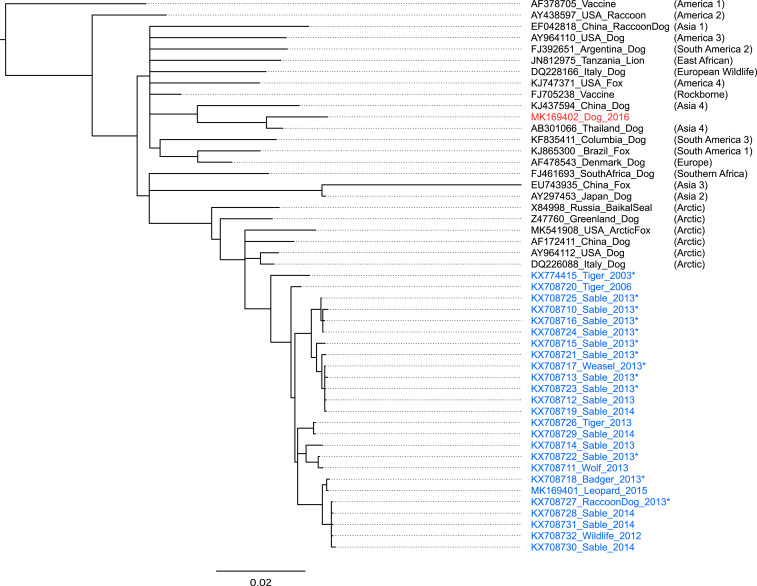
A Bayesian phylogeny generated using hemagglutinin gene sequences from canine distemper viruses (CDVs) detected in Primorskii Krai and a selection of published sequences representing recognized CDV clades (in parentheses). Markov Chain Monte Carlo chains were performed using the software Geneious, version 8.1.8, and the MrBayes plug-in, with the Hasegawa, Kishino, and Yano substitution model. Sequences from Russian wildlife are highlighted in blue, and the Russian dog virus is highlighted in red; details are provided in *SI Appendix*, Table S4. Confidence was increased by concatenating complementary fusion genes where these were available (indicated by *), as a Shimodaira–Hasegawa test indicated a consistent topology for trees based on hemagglutinin and fusion genes (*P* = 0.4622). The phocine distemper virus (KC802221) was used as an outgroup (omitted for scale).

Phylogenetic trees generated using these sequences showed that tiger, leopard, and other wildlife viruses clustered together with published sequences from the Arctic-like clade (19), whereas the dog virus was genetically distinct, aligning within the Asia-4 clade alongside sequences from Thailand and China (20) (Fig. 1). Additional sequences encoding fusion glycoproteins (F-genes, 1,954 bp) from 13 of these animals added topological support to the trees generated. The genetic data show that closely related viruses are infecting a wide range of wild carnivore hosts across a wide geographic area over a prolonged time period, but there is no evidence of a relationship to viruses circulating in domestic dogs across the broader region. Indeed, the results from the single dog suggest that a distinct epidemiological cycle is occurring there. However, with more extensive sequence data, it is possible that we would detect Arctic-like viruses also circulating in dogs, particularly in more remote areas. The key point is that, even without a full understanding of CDV in dogs, we now have sufficient evidence to suggest that interventions that focus only on domestic dogs would not be effective at preventing infection of tigers because of the role of wildlife.

Indeed, together with the serological data, these results are consistent with an interpretation that wildlife are likely to be key hosts contributing to CDV maintenance across the Russian Far East and an important source of CDV infection for tigers in this area. There are several potential routes of CDV transmission from wild carnivores to tigers. The recent detection of CDV in a raccoon dog killed by a tiger in southern Primorskii suggests that predation is one such plausible route. Susceptible species (including Siberian weasels, sable, raccoon dogs, and wild boar) are occasionally observed attending tiger kills, providing a possible opportunity for indirect transmission. Exposure of free-ranging wild boars in Japan and limited CDV replication in lymphoid cells of experimentally infected pigs introduce the potential of transmission from noncarnivore prey (21, 22).

Armed with this evidence, our second objective was to investigate the feasibility of interventions targeted at wildlife. The lack of an oral delivery system for a CDV vaccine renders the prospects of controlling infection in a wild reservoir in this ecosystem logistically untenable. In these circumstances, the only practical means of reducing the extinction threat would be to consider individually vaccinating sufficient numbers of tigers to ensure populations can withstand future outbreaks.

As an essential first step to evaluating the feasibility of this approach, we confirmed that vaccinated tigers could neutralize the CDV strain circulating in tiger habitat. Conventional modified live vaccines have been found to be safe for administration to captive tigers, and elicit a measurable humoral immune response (23). However, the use of these products in field situations requires assurance of their capacity to induce responses that neutralize the wild CDV strain infecting tigers. Vaccine-induced antibodies must bind to the external H and F glycoproteins of the wild viral strain to protect otherwise-susceptible tigers. By using a neutralization assay based on vesicular stomatitis virus (VSV) pseudotypes (24) bearing the external glycoproteins of the Russian viral strain (KX708722), we showed that serum from tigers vaccinated with a modified live vaccine (Nobivac DP; Merck) could neutralize the local strain, providing a credible means of protecting individual tigers from disease (*SI Appendix*, Fig. S1).

**Fig. 2.**
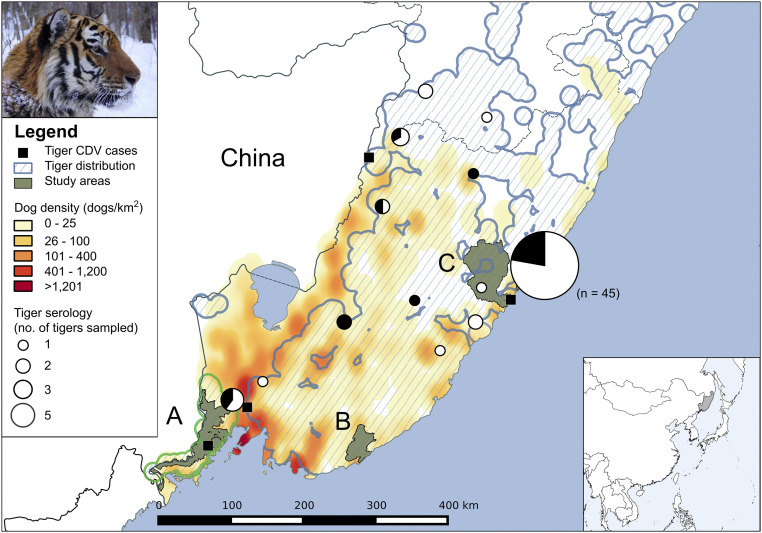
Estimated densities of domestic dogs in the Russian territory of Primorskii Krai represented as a heatmap shaded according to the number of dogs per 1 km^2^ based on a smoothing radius of 25 km. Tiger distribution based on snow-tracking data are represented by the blue-hatched polygons. Serological data from live sampled tigers are indicated as pie charts that are scaled based on sample size (with the exception of Sikhote-Alin Biosphere Zapovednik [SABZ] where sample size is included as text), indicating proportions of positive samples with canine distemper virus (CDV) neutralizing antibodies (black) and negative samples (white). Confirmed cases of CDV-infected tigers are indicated by black squares. The tiger population around Land of the Leopard National Park (LLNP) is highlighted in bright green. Study areas LLNP (*A*), Lazovskii Zapovednik (*B*), and SABZ (*C*) are shaded in dull green.

Contemporary CDV vaccines must be delivered by injection, which places a practical constraint on the rate of vaccine administration for a species as rare and cryptic as the Amur tiger. This would require time-consuming capture operations or, theoretically, delivery through a remote darting system (25), with administration limited to a very small number of tigers each year. However, low-coverage vaccination approaches have been shown to be effective during outbreaks of rabies in controlling spread and preventing the extinction of Ethiopian wolf packs (26). Our previous individual-based stochastic susceptible–infected–recovered/dead (SIRD) model with demography demonstrated the high probability of extinction of Amur tigers over the next 50 y in small isolated populations in the absence of CDV control strategies (8). We adapted this population viability analysis to assess the effectiveness of achievable low coverage vaccination strategies to reduce the 50-y extinction probability of the population of Amur tigers in the vicinity of the Land of the Leopard National Park (LLNP) (Fig. 2), using a demographic susceptible–vaccinated–infected–recovered/dead (SVIRD) model. Although the LLNP population is small and genetically isolated, it is of high conservation value as a source for recolonizing formerly occupied habitat in northeast China (27). The SVIRD model predicted that while reactive strategies (triggered by the detection of affected tigers) had little effect on extinction probability, a proactive approach (vaccinating small numbers of tigers each year without spatial selection) could substantially reduce the risk of extinction. Using realistic model parameters based on the findings of the current study and from the literature, annual vaccination of two tigers per year reduced the 50-y extinction probability of the LLNP population from 15.8 to 5.7% at a mean annual cost of less than $30,000 (Table 3).

**Table 3.** Summary of mean 50-y extinction likelihood values for the tiger population in the vicinity of the LLNP, based on 1,000 simulations of a stochastic individual-based population viability model

Vaccination scenario	50-y extinction likelihood, %	Mean no. of tigers vaccinated	Estimated cost, USD
Control (without CDV)	1.8	0	Not applicable
Control (with CDV)	15.8	0	Not applicable
Reactive strategy	12.8	9.42	287,465
Annual strategy	5.7	100	1,483,724

Costs of implementing a reactive vaccination strategy (vaccinating two tigers when outbreaks are detected) and an annual vaccination strategy (vaccinating two tigers per year) are estimated in US dollars over the 50-y study period based on the mean number of tigers vaccinated and the costs of equipping and supplying capture teams (*SI Appendix*, Table S7).

A culture of risk aversion has led to a reluctance among wildlife authorities to consider wildlife vaccination as a means of preventing the extinction of endangered populations. Specifically, a now discredited hypothesis that attributed the extinction of African wild dogs (*Lycaon pictus*) in the Serengeti to a rabies vaccination intervention (11) has had far-reaching influence on conservation policy, with wildlife vaccination often rejected as a potential conservation tool (10). Instead, wildlife managers have chosen to rely on vaccination of domestic reservoirs as the preferred strategy to control pathogen spillover into endangered species. Our findings counter the assumption that domestic dogs must be acting as CDV reservoirs for Amur tigers and should dispel the perception that domestic dogs are the principal source of infection of CDV. Future policy must be based on a rational interpretation of epidemiological evidence augmented by ex situ assessments of vaccine safety and efficacy, and the use of modeling in the design of feasible delivery strategies for a given context.

## Materials and Methods

### Dog Demography Surveys.

Extreme winter temperatures in Primorskii Krai, which regularly dip below −15 °C, limit opportunities for dogs to subsist without human care, and as a result feral dogs are limited or absent, particularly outside urban centers. For this reason, this study concentrated on owned dogs, as these constitute the vast majority of dogs within the territory. Surveys focused on rural settlements within 25-km buffers surrounding the boundaries of the protected areas LLNP (N42.5°, E130.4° to N43.8°, E131.7°), Lazovskii Zapovednik (N42.9°, E133.7° to N43.4°, E134.2°), and Sikhote-Alin Biosphere Zapovednik (SABZ) (N44.8°, E135.7° to N45.7°, E136.8°). Study areas contained 84 settlements according to the 2010 population census (28). A random selection of 26 rural settlements was generated for demography surveys (including 16/64 around LLNP, 7/16 around Lazovskii, and 3/4 around SABZ). Settlements were classified based on the number of residents present in 2010 as villages (≤1,000 people), towns (>1,000 people, ≤10,000), large towns (>10,000 people, ≤100,000), or cities (>100,000 people).

Target household sample sizes were selected using the “pwr” package in R (29). A minimum sample size of 389 households per study area was chosen using a two-tailed test to detect differences in proportional responses to questions of 10%, with an expected response of 50% (which maximizes required sample size), at a power of 80% and a 95% significance level.

A questionnaire was developed based on published guidelines (30) to collect information on dog ownership patterns and demography. Questionnaires were designed and optimized during pilot surveys in July 2012 and November 2012. Interviews were conducted in Russian during visits to all 26 settlements in November 2012, June 2013, and October 2014. Interviewers visited all residences in villages, explained project objectives to householders, and requested their compliance in completing the surveys irrespective of whether they owned dogs. In towns and large towns, it was impractical to survey all residences. In these settlements, a subset of residences was randomly generated using published methods (31).

Interviews consisted of a list of questions that were completed in a semistructured manner designed to obtain data at the level of the household, and on individual dogs currently living in the residence. Household data included the number of human residents, the number of dogs currently owned, and the number of dogs owned 10 y previously. Dog-related questions included vaccination history, frequency of movement beyond the settlement (never, rarely, at least annually, at least monthly, at least weekly), and freedom to roam unsupervised beyond the property boundaries (never, rarely/sometimes, part of the day, or all day).

A total of 2,576 rural questionnaires were completed across the 26 study settlements, with a mean coverage per settlement of 62.8% (SD: 54.2%). The study areas contained no cities; therefore, a simplified urban questionnaire was used in the city of Ussuriysk (situated less than 20 km from LLNP) to collect data describing dog ownership in urban areas. A total of 1,461 urban questionnaires were completed, focusing on residents interviewed along major thoroughfares, transport hubs, and outside popular groceries. Interviewees were asked to provide their settlement of residence, and the number of people and dogs within their household.

Human population growth was estimated for each study settlement using the census figures from 2002 and 2010. These growth estimates were used to extrapolate the 2010 census figures in order to obtain estimated population sizes at the start of the study on November 6, 2012. Ratios of humans to dogs in surveyed households were used to estimate total numbers of dogs in each settlement, and also used as the basis for extrapolating numbers of dogs across the territory of Primorskii Krai at the start of the study in 2012. For this purpose, the distribution of human-to-dog ratios for settlements of each size category was obtained by resampling the data using a subsample of 100 households in each settlement size category, through 1,000 bootstrap replicates. These were fitted to a gamma distribution, which was then used to estimate the number of dogs in all settlements in each study area, and throughout Primorskii Krai, across 1,000 replicates.

### CDV Exposure History.

Samples from tigers and other large-bodied wild carnivores were obtained from the archives of the Wildlife Conservation Society (WCS) (Bronx, NY) and were collected from wild carnivores captured for research purposes or in response to incidents of human–carnivore conflict in Primorskii Krai (123 animals), neighboring Khabarovskii Krai (six animals) and Amurskaya Oblast (one animal) between November 1992 and November 2014. These included samples from 40 tigers described previously by Goodrich et al. (32).

Serum samples from small-bodied mesocarnivores were obtained from animals captured specifically for this project, and from archived material. Captures took place in Lazovskii Zapovednik (during May 2013, and October/November 2013), and SABZ (April/May 2014), using folding cage traps (Tomahawk and Havahart). Archived material included samples collected in LLNP during 2007 to 2008 (by the Federal Scientific Center of East Asian Terrestrial Biodiversity, Far Eastern Branch of Russian Academy of Sciences, Vladivostok; and the NIH/National Cancer Institute, Frederick, MD), Lazovskii Zapovednik during 2008 to 2009 (by the Zoological Society of London), and from SABZ between 2005 and 2011 (by WCS). Wildlife samples were stored at −20 °C for up to 8 y prior to export, after which they were transferred to −80 °C, and shipped using dry ice for analysis.

Serum samples from dogs were collected in conjunction with rural questionnaire surveys and from 11 additional communities in LLNP, from dogs whose owners provided informed consent.

Serum from vaccinated tigers was generously donated by E. Ramsay, College of Veterinary Medicine, University of Tennessee, Knoxville, TN (23).

Antibody titers were measured using virus neutralization at the Washington Animal Disease Diagnostic Laboratory at Washington State University (tigers and other large-bodied wild carnivores), and by the Veterinary Diagnostic Services at the University of Glasgow (mesocarnivores and domestic dogs). The methods used in both laboratories were based on Appel and Robson (33). Final antibody titers were calculated using the Spearman–Karber method (34). Titers ≥1:16 were considered to be positive to reduce numbers of false positives due to nonspecific neutralization at lower titers. Only serum neutralization titers from animals greater than 4 mo old were included in data analyses, to exclude possible maternally derived antibodies. Seroprevalence was considered to be the number of animals testing positive, divided by the total number of animals tested. The R package “prevalence” (35) was used to calculate 95% binomial confidence intervals for all seroprevalence estimates.

### CDV Molecular Analyses.

Domestic dog samples were obtained from two sources: 1) clinically healthy dogs sampled during household surveys, and 2) dogs presented for treatment at veterinary clinics. Household samples were collected from all dogs whose owners consented to sample collection during household surveys in the study areas of LLNP, Lazovskii Zapovednik, and SABZ. Nasal swabs were preserved in 300 µL of RNAlater stabilizing reagent (Qiagen), and whole blood was collected from the cephalic vein into Vacutainers containing EDTA as an anticoagulant (Becton, Dickinson and Company). All samples were then frozen (at −20 °C or lower) until analysis.

State and private veterinarians in rural and urban areas agreed to participate in the collection of clinical samples from sick dogs using a broad case definition, to maximize the chances of detecting CDV infections. Veterinarians were requested to collect conjunctival and nasal swabs in RNAlater from all dogs displaying any combination of upper respiratory disease, oculonasal discharge, gastrointestinal disease, and/or neurological signs. Participating veterinarians were based in the city of Vladivostok, town of Arsenev, and the districts of Lazovskii, Ussuriyskii, Nadezhdinskii, Khankayskii, Khasanskii, Dalengorskii, and Partizanskii.

Tissue samples were obtained from small-bodied wild carnivores (mesocarnivores) with the assistance of state hunting inspectors in the districts of Terneiskii, Lazovskii, and Pozharskii. These inspectors contacted local fur trappers authorized to capture fur-bearing species during the winter hunting seasons of 2011/2012, 2012/2013, and 2013/2014. Additional samples were obtained from dead mesocarnivores encountered opportunistically, including road traffic accidents, or animals found dead in the forest, and from tigers and bears during routine necropsy examinations. Approximately 30 µg of brain tissue from each animal was frozen at −20 °C in 1 mL of RNAlater. In addition, frozen tissue samples and blood products were obtained from the archives of WCS. Blood products were also selected for RNA extraction from animals with measurable titers of serum antibodies of at least 1:16.

Formalin-fixed paraffin embedded (FFPE) blocks of tissue that had previously been analyzed by Seimon et al. (7) were selected for further extraction, with the objective of extending published sequences. Following the animal codes used by Seimon et al. (7), these included the confirmed CDV cases PT61/Pt 2004, Pt 2010-2, and PT56/Pt 2010-3 (which previously yielded the CDV sequences: KC579363 [PT61/Pt 2004; partial H gene], KC579361 [PT61/Pt2004; partial P gene], and KC579362 [PT56/Pt2010-3; partial H gene]), and from suspected case PT90/Pt 2010-1.

### Sanger Sequencing.

In preparation for RNA extraction, samples were centrifuged at 17,562 rcf for 5 min, to facilitate the removal of RNAlater. Tissue samples were macerated in 200 µL of Buffer RLT Plus (Qiagen), with 1% β-mercaptoethanol and 5 µL of 3U proteinase-K, vortexed regularly, and incubated for 1 to 3 h at 56 °C, until completely homogenized. RNA was extracted from tissue homogenates using the AllPrep DNA/RNA Mini Kit (Qiagen) following manufacturer’s instructions. Extraction of RNA from nasal swabs, whole blood, blood clots, and scat samples was performed using the QIAamp Cador Pathogen kit (Qiagen), using manufacturer’s instructions.

All extracts were initially screened by qPCR for a 114-bp fragment of the P-gene based on previously described protocols (36). Reactions were performed using the Qiagen OneStep RT-PCR kit (Qiagen), and primers CDVF4 and CDVR3, and a TaqMan probe reporting in the FAM channel (*SI Appendix*, Table S5). All reactions included a negative control, and a synthetic CDV sequence as a positive control. Reactions were performed using a Bio-Rad Minopticon Cycler through 45 cycles, with a transcription step of 20 min at 50 °C, and an annealing step of 30 s at 60 °C. Wells with characteristic amplification curves of cycle threshold <38 were considered to be positive. Samples that tested positive in at least one of three wells then underwent additional rounds of RT-PCR amplification, using primer sets for a 429-bp fragment of P-gene (using primers Morb1/Morb2; *SI Appendix*, Table S5), and a 291-bp fragment of the H-gene (with primers TSCDVH2-F/TSCDVH3-R; *SI Appendix*, Table S5). Reactions were performed using a Bio-Rad Minopticon Cycler through 45 cycles, with a transcription step of 30 min at 50 °C, and an annealing step of 60 s at 45 °C. Products were separated by electrophoresis on a 1.5% agarose gel, and all extracts that produced bands of the expected molecular weight using both sets of primers were prioritized for further amplification and sequencing.

Prioritized extracts were amplified by RT-PCR, using four sets of primers that covered the entire H-gene, modified from Müller et al. (37) (1F/1R, 2Farctic/2Rarctic, 3Farctic/3R, 4Farctic/4R; *SI Appendix*, Table S5). Reactions were performed using a Bio-Rad Minopticon Cycler through 45 cycles, with a transcription step of 30 min at 50 °C, and an annealing step of 55 s at 45 °C. Products were separated by electrophoresis on a 2.0% agarose gel, and bands of the expected molecular weight were cleaned using the ExoSAP-IT reagent (Affymetrix) and directly sequenced in the forward and reverse directions (Genewiz).

For positive samples that did not yield full-length H-gene consensus sequences, DNA cloning techniques were employed to obtain additional sequences. Further DNA cloning was used to obtain near full-length F-gene sequences for all samples from which full-length H-genes were sequenced. RNA extracts were used to prepare cDNA for DNA cloning using the ProtoScript First Strand cDNA Synthesis Kit (New England Biolabs) following manufacturer’s protocols, excluding the optional RNA denaturation step. The first strand DNA product was then amplified using Q5 High-Fidelity DNA Polymerase (New England Biolabs) and primers RusCDV5primUTR and RusCDV5primUTR (H-gene) and CDV5primUTR and CDV3primUTR (F-gene) (*SI Appendix*, Table S5) in a VERITI 96-well fast thermocycler (Applied Biosystems) through 35 cycles (with a denaturation step of 98 °C for 10 s, an annealing step of 50 to 72 °C for 30 s, and an extension step of 72 °C for 20 s). A second round of DNA amplification was performed using primers AmurtigercdvHsalF and AmurtigercdvHnot1R (H-gene) and cdvFsal1Fwd and cdvFnot1R (F-gene) (*SI Appendix*, Table S5), to tag PCR products with recognition sites for the SalI and NotI restriction enzymes (New England Biolabs). DNA fragments were then ligated into the pVR1012 eukaryotic expression vector (Vical) and transformed into *Escherichia coli*. Cloned DNA was amplified, purified, and directly sequenced in the forward and reverse direction using a Pacific Biosciences PacBio RS II sequencer (operated by GATC Biotech).

### Next-Generation Sequencing.

#### Sectioning.

A 10-y-old FFPE brain tissue sample was available for analysis (from tiger PT61/Pt 2004). The 20 × 20-µm sections were cut with a microtome in strictly sterile conditions. All areas including the microtome were treated with 10% bleach and RNA-Zap prior to sectioning of each sample. Excess paraffin was removed with a fresh scalpel (new scalpels were used for each biopsy sectioned). Sections were placed in Eppendorf tubes (four per tube) and 1 mL of xylene was added to each tube in a fume hood. A Qiagen FFPE RNeasy kit was then used to extract RNA according to the manufacturer’s instructions following the xylene extraction protocol. Two protocol deviations were made. First, the DNase step was reduced from 15 to 5 min in order to minimize the loss of RNA, and second, RNA was concentrated by running the whole sample through the same minielute column. RNA was eluted in 14 µL and frozen at −80 °C prior to use for library preparation.

#### cDNA synthesis.

cDNA synthesis was carried out with random hexamers using SuperScript III (Invitrogen) according to the manufacturer’s instructions. Double-stranded cDNA was produced using a NEBNext Second Strand Synthesis kit (New England Biolabs) according to the manufacturer’s instructions.

#### Library preparation.

A KAPA DNA library preparation kit (KAPA BioSciences) was used to prepare the cDNA for Illumina sequencing, with the following modifications to standard protocol. In order to minimize sample loss, a “with-bead” approach was used—AMPureXP beads were retained during all library preparation steps (38). To compensate for the low sample input, 150 fmol of NEBNext adapter (New England Biolabs) were used for ligation (a 1,000-fold reduction from standard protocol). Adapter-ligated DNA was amplified on an ABI 7500 real-time PCR cycler, using a real-time library amplification kit (KAPA BioSciences). Real-time amplification enables the reactions to be stopped after the optimum number of PCR cycles, avoiding over- or under-amplification of library DNA. Index tags were added using NEBNext multiplex oligos. Library DNA concentration was assessed with the Qubit 2.0 fluorometer, and an Agilent 2200 TapeStation was used to verify the final size profile of amplified library DNA and ensure no carryover of primer dimers. Up to six DNA libraries with appropriate index tags were pooled, and 2 × 150-nt paired-end sequence datasets were generated on an Illumina MiSeq using 300-cycle v2 reagents.

#### Bioinformatic analysis.

Raw fastq files were examined for quality using FastQC and adaptors and low-quality sequence removed. A de novo assembly was carried out following removal of mammalian DNA sequences using Spades (39), and Tanoti (https://www.bioinformatics.cvr.ac.uk/Tanoti/) was then used to map sequence reads to the CDV contig and to the nearest reference sequence.

### Phylogenetic Analysis.

Sequences were aligned using the MUSCLE algorithm within the software Geneious (version 8.1.8) and were edited manually. Appropriate nucleotide substitution models were identified using jModeltest 2, version 2.1.8 (40, 41), with best-fit models selected based on lowest Akaike information criterion scores. Phylogenetic trees were constructed for the H-genes and F-genes using Bayesian inference. The Shimodaira–Hasegawa test was used to confirm that there was no significant difference between the topology of the H-gene and F-gene trees (*P* = 0. 462), and the H-gene and F-genes were concatenated to increase confidence in the construction of the final tree. The final tree was based on the GTR+G nucleotide substitution model and was constructed using the Geneious MrBayes plug-in (Version 3.2.6), with 1,100,000 iterations, subsampling every 200 trees and discarding 25% of samples as burn-in (42).

### Pseudotype Microneutralization.

The recombinant VSV in which the glycoprotein (G) gene has been deleted (VSVΔG) and replaced with firefly luciferase (*luc*) has been described (43, 44) and was kindly provided by Michael Whitt, University of Tennessee Health Science Center, Memphis, TN. An initial stock of VSVΔG*luc* bearing VSV-G was used to infect 293T cells transfected with the VSV-G expression vector pMDG (45). VSVΔG*luc* (VSV-G) pseudotypes were recovered, titrated on 293T cells, and used to prepare a working stock of VSVΔG*luc* (VSV-G) pseudotypes. To prepare CDV H and F expression constructs, viral RNA was prepared from mesocarnivore tissue samples (AllPrep DNA/RNA Mini kit; Qiagen), used to prepare first-strand cDNA (Transcriptor First-Strand cDNA Synthesis Kit; Roche), and then used as template in PCRs with the following primers: CDV H Sal, 5′-GTC​GAC-ACC-ATG​CTC​CCC​TAC​CAA​GAC​AAG​GT-3′; CDV H Not, 5′-GGG​CGG​CCG​C-TTA​ACG​GTT​ACA​TGA​GAA​TCT​TA-3′; CDV F Sal, 5′-GGG​TCG​AC-ACC-ATG​CAC​AGG​GGA​ATC​CCC​AAA​AG-3′; and CDV F D633 Not, 5-GGG​CGG​CCG​C-TTG​CTA​GCG​TCT​TTT​ACA​ACA​GTA​AAT​CAG​CA-3′ (Expand High Fidelity PCR system; Roche). Amplifications were performed with the following thermocycling conditions: denaturation at 94 °C for 5 min, followed by 35 cycles of 94 °C for 30 s, annealing at 50 °C for 60 s and extension at 72 °C for 120 s, with a final extension at 72 °C for 10 min. Products were digested with the enzymes SalI and NotI and cloned into the eukaryotic expression vector VR1012 (Vical). Canine SLAM-F1 (dogSLAM) was amplified from total RNA prepared from mitogen-stimulated canine peripheral blood mononuclear cells using PCR (Q5 High-Fidelity DNA Polymerase; New England Biolabs) and the primers dogSLAM Bgl, 5′-GCT​CAG​ATC​TGA​GAG​CTT​GAT​GAA​TTG​CCC​AG-3′, and dogSLAM Sal, 5′-GCT​CGT​CGA​CGC​TCT​CTG​GGA​ACG​TCA​C-3′. Amplifications were performed with the following thermocycling conditions: denaturation at 98 °C for 30 s, followed by 30 cycles of 98 °C for 10 s, annealing at 65 °C for 30 s and extension at 72 °C for 60 s, with a final extension at 72 °C for 2 min. The amplified cDNA was cloned into the pDisplay eukaryotic expression vector (Life Technologies) using BglII and SalI. The nucleic acid sequences of all amplified cDNAs were determined externally by Sanger dideoxy chain termination sequencing (*LIGHTrun* Sequencing Service; GATC Biotech AG). All oligonucleotide primers were obtained from Integrated DNA Technologies.

To prepare target cells for the VSV-ΔG(CDV) pseudotypes, HEK293 cells were transfected with pDisplay-dogSLAM using linear polyethylenimine, MW 25,000 (Polysciences), and selected in complete medium supplemented with 800 µg/mL G418. The stably transfected 293-dogSLAM cells were expanded and the surface expression of SLAM confirmed by flow cytometry using rabbit polyclonal anti-H (Sigma) followed by phycoerythrin (PE)-conjugated goat anti-rabbit IgG (Sigma) on a BD Accuri flow cytometer (BD) (24).

Neutralizing antibodies were determined by pseudotype virus neutralization assay (4). A total of 2 × 10^4^ 293-dogSLAM cells was plated into each well of a 96-well white flat-bottomed plate (Culturplate-96; Perkin-Elmer). Fourfold serum dilutions were prepared in triplicate in complete medium ranging from 1:8 to 1:32,768. The diluted serum samples were then added to the 293-dogSLAM cells followed by 2.5 × 10^3^ TCID50 of VSVΔG(CDV) pseudotype. Plates were incubated for 48 to 72 h at 37 °C, at which time luciferase substrate was added (Steadylite plus; Perkin-Elmer) and the signal analyzed on a Microbeta 1450 Jet luminometer (Perkin-Elmer). Antibody titers were calculated by interpolating the point at which there was a 90% reduction in luciferase activity (90% neutralization).

### Vaccination Model.

A stochastic population viability model developed by Gilbert et al. (8) was extended to assess the feasibility of vaccination strategies to reduce the 50-y extinction probability of the small isolated population of Amur tigers in the vicinity of LLNP. The extended model includes a vaccinated state, giving an SVIRD model with five possible infection states for each individual tiger—susceptible, vaccinated, infected, recovered and dead, respectively—on top of their demographic characteristics (described in ref. 8 and summarized below). The model was discrete time (operating on a 2-wk time step), stochastic, and individual-based and was developed in the object-oriented programming language Ruby, version 2.3.0.

Following ref. 8, we assumed a closed population of tigers allotted to two social categories: territory holders that retained ownership of their territory until death, and nonterritory holders that move throughout the landscape until encountering a vacant territory. Available habitat was considered sufficient to support six male territories and nine female territories. Only territory holders were permitted to reproduce. Parameters used for reproduction and survival are summarized in Gilbert et al. (8).

The model was allowed a burn-in period of 40 y to achieve a dynamic equilibrium in population size, followed by a 50-y study period, whereby CDV infection is introduced into the population and vaccination commences. Only simulations where population size at the start of the 50-y study period fell in the range of 15 to 27 tigers were used to assess 50-y extinction probability. Populations were assessed over 1,000 simulations and 50-y extinction probability calculated as the percentage of simulations where population size declined to zero.

Tigers contracted infections in one of three ways: 1) when predating infected dogs, 2) when predating infected wild carnivores, and 3) during social contact with infected tigers. The probability of a susceptible tiger acquiring infection in a given time step is given by $1-{\left(1-p\right)}^{c}$, where *c* is the number of effective contacts per time step (i.e., those where transmission would occur should the contact be with an infectious individual), and *p* is the prevalence of CDV shedding among those contacts, which may be tigers, other wild carnivores, or domestic dogs. Infection parameters are summarized in *SI Appendix*, Table S6. The number of predated dogs and wild carnivores was generated from a Poisson distribution. To reflect uneven distribution of dogs, predation was restricted to 80% of territories where human settlements were estimated to occur. All nonterritory holders were considered capable of dog predation as they were assumed to disperse more widely including into areas of modified habitat. Predation of wild carnivores was limited to time steps between early April and early November, when most species are more active. Tigers maintain territories that largely exclude members of the same sex; therefore, individuals could only contract CDV from members of the opposite sex. Dependent cubs automatically contract CDV if their mothers are infected, and do not survive if their mother dies. Mortality from CDV infection was estimated as 35% for all ages of tiger, and surviving tigers recovered after 45 d.

The prevalence of domestic dogs infected with CDV was estimated using a catalytic model based on the age-stratified serological data collected from unvaccinated dogs in the study communities (46). The number of seropositive and seronegative dogs in each age category *k* is given by *n*_pos_(*k*) and *n*_neg_(*k*), respectively, where the *k*th age category contains animals in the age range *k* − 1 to *k* years. *n*_pos_(*k*) represents the number of dogs surviving infection, and can be used to estimate the number of animals that have been infected *n*_inf_ (*k*) for a given CDV mortality ratio *M* (defined as the proportion of infected dogs that die from the infection) given by the following:

$$n_{\text{inf}}\left(k\right)=\frac{n_{\mathrm{pos}}\left(k\right)}{\left(1-M\right)}.$$

The proportion of dogs infected by a given age is assumed to follow a catalytic model, i.e., the population is assumed to be subject to a constant force of infection, *λ*. We therefore assume that the proportion *p*_inf_ (*k*) of dogs infected by the time they reach age category *i* is given by the following:

$$p_{\inf}\left(k\right)=1-e^{-\lambda a_{k}},$$

where *a*_*k*_ is the midpoint of the *k*th age category. The likelihood contribution of each age category *i* was then given by the following:

$$n_{\inf}\left(k\right)\log\left(p_{\text{inf}}\left(k\right)\right)+n_{\mathrm{neg}}\left(k\right)\text{log}\left(1-p_{\inf}\left(k\right)\right).$$

An estimate of *λ* was derived using the maximum likelihood with CIs obtained using the likelihood ratio test.

Assuming that CDV infections did not occur seasonally, mean infection prevalence *P* was estimated assuming a mean period of infection *I* of 21 d (47):

$$P=\lambda \frac{I}{365}.$$

Estimated *λ* was 0.134 (CI: 0.116 to 0.154) cases per dog per year, which equates to a mean prevalence of 0.77% assuming *M* of 0.35 (47).

Control scenarios were run both with and without CDV to determine baseline extinction probabilities. Two vaccine scenarios were used to simulate alternative vaccination parameters. These included an annual vaccination program, in which two tigers were vaccinated each year. A reactive vaccination scenario was also used, where vaccine was only delivered after the detection of an outbreak and were given to two tigers. The probability of outbreak detection was given by the following:

$$1-{\left(1-\tau \right)}^{c},$$

where *c* is the expected number of tiger deaths as a consequence of infection and *τ* is the probability of finding a dead tiger in a condition where diagnosis would be possible. The value of *τ* was estimated to be 0.25, based on the fate of tigers identified in camera traps in the SABZ between 2006 and 2013 (48).

Vaccines were administered randomly to adult tigers irrespective of territorial status, or stage of infection or immune status. For infected tigers, vaccination did not act therapeutically; consequently, their infection status remained unchanged. Vaccines were assumed to be 90% effective (inducing protective immunity in 90% of individuals that received a single dose) and to provide lifelong protection from infection with CDV.

Mean costs of vaccination scenarios were estimated over 50 y based on the mean number of tigers vaccinated across all 1,000 simulations. Budgets comprised equipment costs to set up each capture team, and the mean costs of salaries, supplies, living expenses, and fuel per tiger captured (*SI Appendix*, Table S7). All equipment was replaced every 10 y to simulate regular wear and tear. Capture effort per tiger was based on Goodrich et al. (49), which describes 19 tigers captured over 12,287 trap nights between 1992 and 1998. Assuming one capture team can monitor a trap line of 15 snares, one tiger would be captured every 43.1 d. With captures timed for two 43-d trapping seasons in the spring and autumn, it would be reasonable for one capture team to capture and vaccinate a mean of two tigers per year. Costs were estimated over the 50-y study period, and effects of inflation were excluded.

## Supplementary Material

Supplementary File

## Data Availability

Spreadsheets, model code, and RNA sequence data have been deposited in Enlighten: Research Data (University of Glasgow data repository) (50) and the GenBank National Center for Biotechnology Information Nucleotide database [accession nos. KX708710–KX708745 (51–85), KX774415 (86), MK169401 (87), and MK169402 (88)]. All study data are included in the article and *SI Appendix*.

## Change History

December 15, 2020: The license for this article has been updated.
