# Supplementary material for: Distemper, extinction, and vaccination of the Amur tiger
Source: Proc Natl Acad Sci U S A. 2020 Nov 23;117(50):31954–62. doi: 10.1073/pnas.2000153117 (PMC7749280; doi:10.1073/pnas.2000153117)
Supplement: Supplementary File [file pnas.2000153117.sapp.pdf]

PNAS
www.pnas.org

Supplementary Information for

Distemper, extinction and vaccination of the Amur tiger.

Martin Gilbert, Nadezhda Sulikhan, Olga Uphyrkina, Mikhail Goncharuk, Linda Kerley, Enrique Hernandez Castro, Richard Reeve, Tracie Seimon, Denise McAloose, Ivan V. Seryodkin, Sergey V. Naidenko, Christopher A. Davis, Gavin S. Wilkie, Sreenu B. Vattipally, Walt E. Adamson, Chris Hinds, Emma C. Thomson, Brian J. Willett, Margaret J. Hosie, Nicola Logan, Michael McDonald, Robert J. Ossiboff, Elena I. Shevtsova, Stepan Belyakin, Anna A. Yurlova, Steven A. Osofsky, Dale G. Miquelle, Louise Matthews, Sarah Cleaveland.

Martin Gilbert
Email: m.gilbert@cornell.edu

This PDF file includes:

Figures S1 to S2
Tables S1 to S7
SI References

www.pnas.org/cgi/doi/10.1073/pnas.2000153117

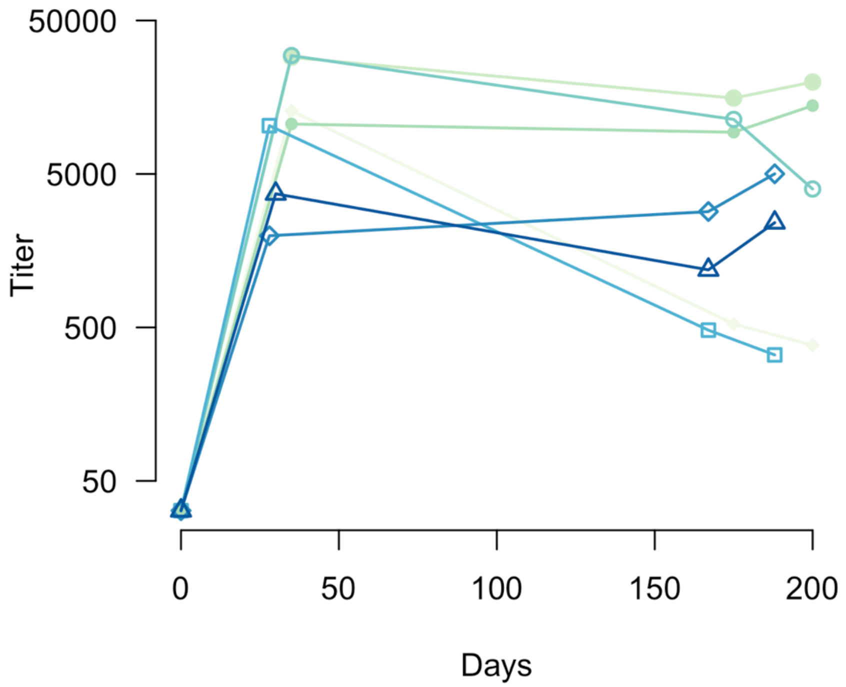

Fig. S1. Neutralizing antibody titers of seven tigers vaccinated with the modified live vaccine Nobivac DP (Merck, Kenilworth, NJ, USA) as assessed using vesicular stomatitis virus (VSV) pseudotypes bearing the H and F glycoproteins of the Russian viral strain (KX708722). Increasing titers of neutralizing antibodies following vaccine administration confirm that sera from vaccinated tigers were able to neutralize the wild strain circulating in tiger habitat.

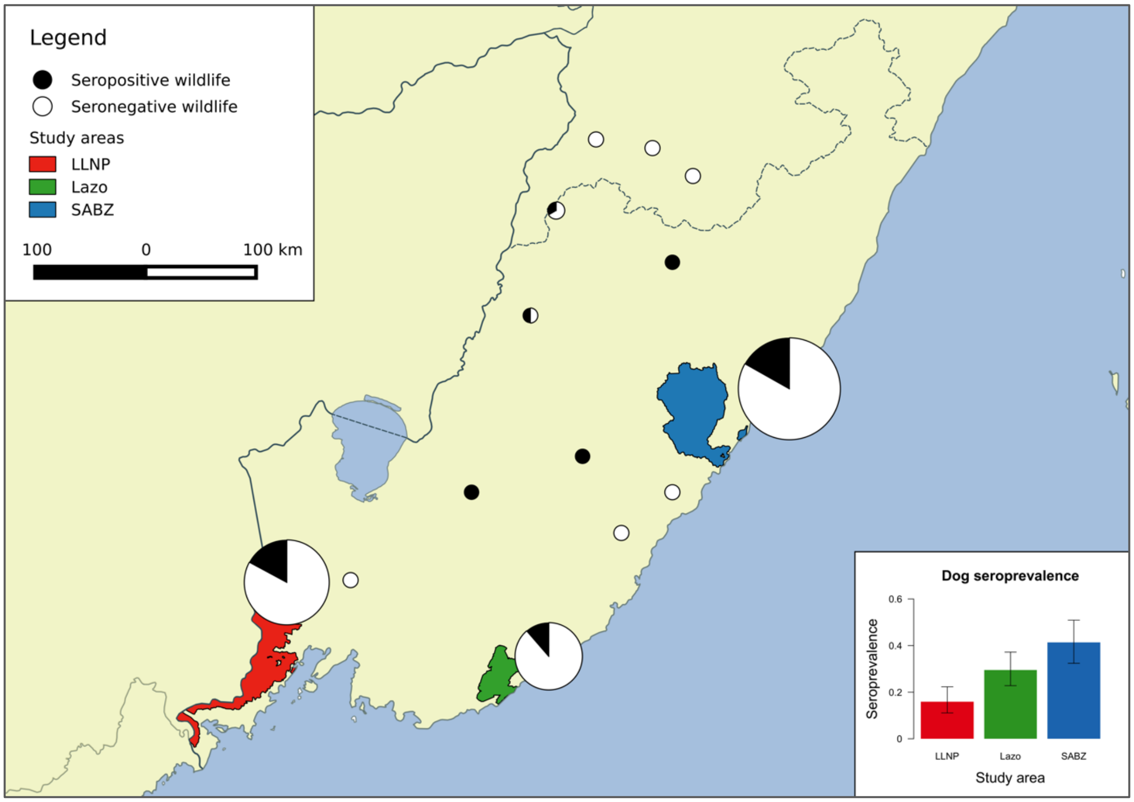

Fig. S2. Map demonstrating the exposure of domestic dogs and wild carnivores to canine distemper virus (CDV) across Primorskii Krai and neighbouring Khabarovskii Krai. Serology results from live-sampled wildlife are indicated as pie charts that are scaled based on sample size, indicating proportions of positive samples with canine distemper virus (CDV) neutralizing antibodies (black) and negative samples (white). These combine data from all sampled Amur tigers, Far Eastern leopards, Eurasian lynx, leopard cats, brown bears, Asiatic black bears, raccoon dogs, red foxes, Asian badgers, sable, Siberian weasels and American mink. Seroprevalence of unvaccinated domestic dogs sampled in the three study areas (Land of the Leopard National, LLNP – red; Lazovskii Zapovednik, Lazo – green; Sikhote-Alin Biosphere Zapovednik, SABZ – blue) are illustrated by the bar chart in the insert panel.

Table S1. Results of virus neutralization analyses against canine distemper virus (CDV) for serum samples collected from wild carnivores in the Russian Far East between 1992 and 2014. Neutralizing antibody titres of 1:16 or higher were considered positive. Animals aged three months or younger have been excluded. Seroprevalence is given as the number of positive samples expressed as a percentage of sample size, with lower and upper 95% binomial confidence intervals (CI). * Samples tested at Washington State University against CDV Onderstepoort strain. † Samples tested at the University of Glasgow using Onderstepoort strain (Bussell derivative).

Species	Positive	Sample size	Seroprev. (%)	Lower CI (%)	Upper CI (%)
Amur tiger *	20	66	30.3	19.9	43.0
Far Eastern leopard *	2	10	20.0	3.5	55.8
Eurasian lynx *	1	7	14.3	0.8	58.0
Leopard cat	2	16	12.5	2.2	39.6
Asiatic black bear *	1	25	4.0	0.2	22.3
Brown bear *	2	20	10.0	1.8	33.1
Raccoon dog †	12	35	34.3	19.7	52.3
Red fox †	0	4	0.0	0.0	60.4
Sable †	0	2	0.0	0.0	80.2
Siberian weasel †	0	2	0.0	0.0	80.2
American mink †	0	2	0.0	0.0	80.2
Asian badger †	2	43	4.7	0.8	17.1

Table S2. Published density estimates (animals/km^2) for the four most abundant mesocarnivore species in Primorskii Krai. Low and high density estimates are based on non-urban settings within published sources, giving preference to Russian sources where available. Density estimates are not available for Asian badgers, so the range quoted refers to the closely related Eurasian badger (*Meles meles*), to which the taxon was formerly considered conspecific. Density estimates are extrapolated across the 155,000 km^2 distribution of the Amur tiger (4) to produce low and high population estimates.

Species name	Low density estimate	High density estimate	Low population estimate	High population estimate	References
Sable *Martes zibellina*	0.04	0.67	6,200	103,850	(Stroganov, 1969)
Asian badger *Meles leucurus*	0.4	1.5	62,000	232,500	(Larivière and Jennings, 2009)
Red fox *Vulpes vulpes*	0.49	1.13	75,950	175,150	(Heydon et al., 2000)
Raccoon dog *Nyctereutes procyonoides*	0.34	0.48	52,700	74,400	(Ward and Wurster-Hill, 1990)
**Total**			**196,850**	**585,900**	

Table S3. Summary of samples tested for the presence of canine distemper virus (using either CDVF4/CDVR3 or Morb1/Morb2 primers), and of haemagglutinin (H) and fusion (F) genes sequenced.

Survey	Species common name	Species scientific name	Sample type	n	CDV +ve	H	F
Household surveys							
	Domestic dog	Canis familiaris	Nasal swab	633	0	0	0
			Whole blood*	205	0	0	0
Clinic surveys							
	Domestic dog	Canis familiaris	Conjunctival swab	75	1	<1**	0
Dead wild carnivores							
	Leopard cat	*Prionailurus bengalensis*	Tissue	30	2	0	0
	Eurasian lynx	*Lynx lynx*	Tissue	4	0	0	0
	Leopard	*Panthera pardus*	Tissue	1	1	1	0
	Tiger	*Panthera tigris*	Tissue	3	1	1	0
	Grey wolf	*Canis lupus*	Tissue	2	1	1	0
	Raccoon dog	*Nyctereutes procyonoides*	Tissue	27	1	1	1
	Red fox	*Vulpes vulpes*	Tissue	9	0	0	0
	Asiatic black bear	*Ursus thibetanus*	Tissue	1	0	0	0
	Sable	*Martes zibellina*	Tissue	518	25	17†	9
	Yellow-throated marten	*Martes flavigula*	Tissue	3	1	0	0
	Siberian weasel	*Mustela sibrica*	Tissue	27	1	1	1
	American mink	*Neovison vison*	Tissue	4	0	0	0
	River otter	*Lutra lutra*	Tissue	3	0	0	0
	Asian badger	*Meles leucurus*	Tissue	5	2	1	1
	Unidentified	Unidentified	Tissue	1	1	1	0
Wildlife surveys							
	Sable	*Martes zibellina*	Nasal swab	2	0	0	0
	Asian badger	*Meles leucurus*	Nasal swab	17	0	0	0
	Leopard cat	*Prionailurus bengalensis*	Nasal swab	8	0	0	0
	Raccoon dog	*Nyctereutes procyonoides*	Nasal swab	10	0	0	0
Archived blood							
	Eurasian lynx	*Lynx lynx*	Serum	1	0	0	0
	Leopard	*Panthera pardus*	Whole blood /clots	2		0	0
	Tiger	*Panthera tigris*	Whole blood /clots	20	1	1	0
	Raccoon dog	*Nyctereutes procyonoides*	Serum	6	0	0	0
	Asiatic black bear	*Ursus thibetanus*	Serum	2	0	0	0
	Brown bear	*Ursus arctos*	Whole blood /clots	1	0	0	0
	Asian badger	*Meles leucurus*	Serum	5	0	0	0
Next generation sequencing							
	Tiger	*Panthera tigris*	Formalin fixed paraffin embedded tissue blocks	4	-	1§	1§
Scat survey							
	Tiger	*Panthera tigris*	Faeces	35	0	0	0
			**TOTAL**	1,664		26†	13

* The 205 whole blood samples were analysed from a subset of the 633 dogs tested during passive surveillance, and so do not represent additional individuals.

** Refers to a partial length sequence.

† Figure includes an H gene from which a gap of 442 base pairs could not be sequenced.

§ Full virus genome obtained from one animal.

Table S4. A summary of complete (COMP) and partial (PART) haemagglutinin gene (H) and fusion gene (F) sequences obtained from carnivores in the Russian Far East. Includes host species, location of origin (KH = Khabarovskii Krai, TY = Terneiskii district, PZ = Pozharskii district, LZ = Lazovskii district, LLNP = Land of the Leopard National Park, VL = Vladivostok), and GenBank accession number.

Species	Study area	H gene	F gene	Accession number (H)	Accession number (F)
Unidentified small carnivore	TY	COMP	-	KX708732	-
Amur tiger	KH	COMP	COMP	KX774415	KX774415
Amur tiger	TY	COMP	-	KX708720	-
Sable	TY	COMP	COMP	KX708721	KX708734
Sable	TY	COMP	COMP	KX708722	KX708735
Grey wolf	TY	COMP	-	KX708711	-
Sable	TY	COMP	-	KX708712	-
Sable	TY	COMP	COMP	KX708713	KX708736
Sable	PZ	COMP	COMP	KX708710	KX708737
Sable	TY	COMP	-	KX708714	-
Sable	PZ	COMP	COMP	KX708723	KX708738
Sable	PZ	COMP	COMP	KX708724	KX708739
Sable	TY	COMP	COMP	KX708715	KX708740
Sable	PZ	COMP	COMP	KX708716	KX708741
Siberian weasel	PZ	COMP	COMP	KX708717	KX708742
Sable	PZ	COMP	COMP	KX708725	KX708743
Asian badger	LLNP	COMP	COMP	KX708718	KX708744
Amur tiger	LLNP	COMP	-	KX708726	-
Raccoon dog	LLNP	COMP	COMP	KX708727	KX708745
Sable	LZ	PART	-	KX708733	-
Sable	LZ	COMP	-	KX708719	-
Sable	LZ	COMP	-	KX708728	-
Sable	LZ	COMP	-	KX708729	-
Sable	LZ	COMP	-	KX708730	-
Sable	TY	COMP	-	KX708731	-
Far Eastern leopard	LLNP	COMP	-	MK169401	-
Domestic dog	VL	PART	-	MK169402	-

Table S5. Primer and probe sequences used in the detection of canine distemper virus. Restriction sequences used in cloning are indicated in red font. Sources refer to 1 = Scagliarini et al. (2007), 2 = Barrett et al., (1993), 3 = modified from Müller et al., (2011) with modifications indicated in blue font, Novel = primers designed for this study.

Primer target	Primer/probe name	Primer sequence	Source
P-gene (partial)	CDVF4	GTCGGTAATCGAGGATTCGAGAG	1
	CDVR3	GCCGAAAGAATATCCCCAGTTAG	
	CDVProbe	6FAM-ATCTTCGCCAGAATCCTCAGTGCT-MGBNFQ	
P-gene (partial)	MorbF	ATGTTTATGATCACAGCGGT	2
	MorbR	ATTGGGTTGCACCACTTGTC	
HA-gene (partial)	TSCDVH2-F	TACTGAGTCCAATTTAGTGGTGTTGCC	Novel
	TSCDVH3-R	CATGAGAATCTTATACGGAC	
HA-gene (complete)	1F	GGGCTCAGGTAGTCCARCAA	3
	1R	CCTCCGGAGAGTGCTGATAA	
	2Farctic	GTGAGACAATTGGGATCAGA	3
	2Rarctic	TGGGTGAGCGACAGGTGTCA	
	3Farctic	TGGGAATCTTTGGGGCAACA	3
	3R	TCCATAATCTGGGATGTTTGAA	
	4Farctic	ATCCCTCATGTGTTATCATT	3
	4R	GACCTCAGGGTATAGAATCTGG	
HA-gene (complete) (cloning)	RusCDV5primUTR	GCTCTGGTAGGAGAGCAATG	Novel
	RusCDV5primUTR	GTCCAATTGAGATGTGTATCATCATACT	
	AmurtigercdvHsalF	GGATGTCGACACCATGCTCTCCTACCAAGATAAGGT	Novel
	AmurtigercdvHnot1R	GGATGCGGCCGCTCAAGGTTTTGAACGGTTACATGAG	
F-gene (complete) (cloning)	CDV5primUTR	ACAAGCCTCATGCACAAGGAAAT	Novel
	CDV3primUTR	GTGACTAGAGTGATTCAGAGTG	
	cdvFsal1Fwd	GGATGTCGACATGCACAAGGAAATCC	Novel
	cdvFnot1R	GGATGCGGCCGCTCAGAGTGATCTTACATAG	

Table S6. Estimated infection parameters used in the population viability model to assess the effectiveness of vaccination strategies to reduce the likelihood of population extinction from CDV. Sources for the estimates used are indicated.

Parameter	Value	Justification
CDV mortality rate	35%	Greene and Apel 2006
Dog prevalence	0.0077	See text
Dogs eaten per year	0.965	Median used in Gilbert et al. 2014
Wild carnivore prevalence	0.034	Median used in Gilbert et al. 2014
Wild carnivores eaten per year	2.76	Median used in Gilbert et al. 2014
Tiger contacts per month (male with female)	2	Goodrich et al. 2010
Tiger contacts per month (female with male)	1	Goodrich et al. 2010

Table S7. Estimated budget for capturing and vaccinating tigers in Primorskii Krai. Budgets are based on 1) the cost of equipping a capture team that is able to vaccinate up to two tigers per year, and 2) the cost of supplying each capture team estimated on a per tiger basis. Costs are estimated in US dollars. * Fuel costs are based on the expense of running a diesel Toyota Land Cruiser with estimated fuel consumption of 15 liters per 100 km and a diesel cost of $0.70 per liter. Vehicles would be required to make two round trips of 100 km per week to keep capture teams supplied while in the field.

1. Setup costs for equipping one trapping team (replace every 10 years)

Item	Unit	Unit cost (USD)	Number of units	Subtotal
Snares	Each	40	40	1,600
Supplies	Total	1	800	800
Dart gun	Each	2,000	1	2,000
Anesthesia equipment	Total	1,300	1	1,300
Holding cage	Each	1,000	1	1,000
Satellite phone	Each	600	1	600
Two-way radio system	Each	300	1	300
Vehicle	Each	25,000	1	25,000
			**Total**	**32,600**

2. Field costs per tiger (i.e. 43 days field time per tiger)

Item	Unit	Unit cost (USD)	Number of units	Subtotal
Daily salary of Russian trapper #1	Per day	45	43	1,955
Daily salary of Russian trapper #2	Per day	45	43	1,955
Daily salary of international trapper	Per day	152	43	6,515
Food (per person)	Per day	5	129	645
Anesthetics	Per tiger	65	1	65
Emergency drugs	Per year	100	1	100
Fuel*	Per km	0	2,457	258
Repairs	Per year	1,500	1	1,500
Satellite phone airtime	Per day	5	43	215
			**Total**	**13,207**

SI References

1. Scagliarini A, Dal Pozzo F, Gallina L, Vaccari F, Morganti L (2007) TaqMan based real time PCR for the quantification of canine distemper virus. *Vet. Res. Commun.* 31 Suppl 1:261–3.
2. Barrett T et al. (1993) Dolphin and porpoise morbilliviruses are genetically distinct from phocine distemper virus. *Virology* 193:1010–1012
3. Müller A, Silva E, Santos N, Thompson G (2011) Domestic dog origin of canine distemper virus in free-ranging wolves in Portugal as revealed by hemagglutinin gene characterization. *J. Wildl. Dis.* 47:725–729.
4. Hebblewhite M et al. (2014) Including biotic interactions with ungulate prey and humans improves habitat conservation modeling for endangered Amur tigers in the Russian Far East. *Biol Conserv* 178:50–64.
5. Stroganov SU (1969) in *Carniverous Mammals of Siberia [Translated from Russian]* (Israel Program for Scientific Translations).
6. Larivière S, Jennings AP (2009) in *Handbook of the Mammals of the World. 1. Carnivores*, eds Wilson DE, Mittermeier RA (Lynx Editions, Barcelona), pp 564–656.
7. Heydon MJ, Reynolds JC, Short MJ (2000) Variation in abundance of foxes (*Vulpes vulpes)* between three regions of rural Britain, in relation to landscape and other variables. *J Zool London* 251:253–264.
8. Ward OG, Wurster-Hill DH (1990) Nyctereutes procyonoides. *Mamm Species* 358:1–5.
9. Goodrich JM et al. (2010) Spatial structure of Amur (Siberian) tigers (*Panthera tigris altaica*) on Sikhote-Alin Biosphere Zapovednik, Russia. *J. Mammal.* 91:737–748.
10. Greene CE, Appel MJ (2006) in *Infectious diseases of the dog and cat*, ed Green CE (Elsevier, St Louis, Missouri), pp 25–41.Third Edit.
11. Gilbert M et al. (2014) Estimating the potential impact of canine distemper virus on the Amur tiger population (*Panthera tigris altaica*) in Russia. *PLoS One* 9:e110811.
